# A law of iterated logarithm for the subfractional Brownian motion and an application

**DOI:** 10.1186/s13660-018-1675-1

**Published:** 2018-04-23

**Authors:** Hongsheng Qi, Litan Yan

**Affiliations:** 1 Department of Mathematics, College of Science, Bengbu University, Bengbu, P.R. China; 2 Department of Mathematics, College of Science, Donghua University, Shanghai, P.R. China

**Keywords:** 60G15, 60G22, 60F17, Sub-fractional Brownian motion, Iterated logarithm, Φ-variation

## Abstract

Let $S^{H}=\{S^{H}_{t},t\geq0\}$ be a sub-fractional Brownian motion with Hurst index $0< H<1$. In this paper, we give a local law of the iterated logarithm of the form

$$\limsup_{s\downarrow0}\frac{ \vert S^{H}_{t+s}-S^{H}_{t} \vert }{ s^{H}\sqrt {2\log^{+}\log(1/s)}}=1, $$

almost surely, for all $t > 0$, where $\log^{+}x=\max{\{1, \log x\}}$ for $x\geq0$. As an application, we introduce the $\Phi_{H}$-variation of $S^{H}$ driven by $\Phi_{H}(x):= [x/\sqrt{2\log^{+}\log ^{+}(1/x)} ]^{1/H}$ $(x>0)$ with $\Phi_{H}(0)=0$.

## Introduction and main results

The quadratic variation and realized quadratic variation have been widely used in stochastic analysis and statistics of stochastic processes. The realized power variation of order $p>0$ is a generalization of the quadratic variation, which is defined as

1.1 $$ \sum_{k=1}^{n} \vert X_{t_{k}}-X_{t_{k-{1}}} \vert ^{p}, $$

where $\{X_{t}, t>0\}$ is a stochastic process and $\kappa=\{ 0=t_{0}< t_{1}<\cdots<t_{n}=t\}$ is a partition of $[0, t]$ with $\max _{1\leq i\leq n}\{t_{i}-t_{i-1}\}\to0$. It was introduced in Barndorff–Nielsen and Shephard [1, 2] to estimate the integrated volatility in some stochastic volatility models used in quantitative finance and also, under an appropriate modification, to estimate the jumps of the processes under analysis. The main interest in these papers is the asymptotic behavior of the statistic (1.1), or some appropriate renormalized version of it, as $n\to\infty$, when the process $X_{t}$ is a stochastic integral with respect to a Brownian motion. Refinements of their results have been obtained in Woerner [3]. A more general generalization to the realized quadratic variation is called Φ-variation, and it is defined by

$$S_{{\Phi}}(X,t,\kappa):=\sum_{k=1}^{n} \Phi \bigl( \vert X_{t_{k}}-X_{t_{k-1}} \vert \bigr), $$

where Φ is a nonnegative, increasing continuous function on ${\mathbb {R}}_{+}$ with $\Phi(0)=0$. Let ${\mathscr {P}}([0,t])$ be a class of all partitions $\kappa=\{0=t_{0}< t_{1}<\cdots<t_{n}=t\}$ of $[0, t]$ with $|\kappa|:=\max_{1\leq i\leq n}\{t_{i}-t_{i-1}\}$. Then the Φ-variation of a stochastic process $\{X_{t}, t>0\}$ is defined as

$$S_{\Phi}(X,t):=\limsup_{\delta\to0} \bigl\{ S_{\Phi}(X,t,\kappa):\kappa\in{\mathscr {P}}\bigl([0,t]\bigr), \vert \kappa \vert < \delta \bigr\} . $$

Consider the function

$$\Phi_{H}(x)= \bigl(x/\sqrt{2\log^{+}\log^{+} (1/x )} \bigr)^{1/H},\quad x>0 $$

with $\Phi_{H}(0)=0$ and $0< H<1$, where $\log^{+}x=\max\{1,\log x\}$ for $x>0$. When *X* is a standard Brownian motion *B*, Taylor [4] first considered the $\Phi_{1/2}$-variation and proved $S_{\Phi _{1/2}}(B,t)=t$ for all $t>0$. Kawada and Kôno [5] extended this to some stationary Gaussian processes *W* and proved $S_{\Phi_{1/2}}(W,t)=t$ for all $t>0$ by using an estimate given by Kôno [6]. Recently, Dudley and Norvaiša [7] extended this to the fractional Brownian motion $B^{H}$ with Hurst index $H\in(0,1)$ and proved $S_{\Phi _{H}}(B^{H},t)=t$ for all $t>0$. More generally, for a bi-fractional Brownian motion $B^{H,K}$, Norvaiša [8] showed that $S_{\Phi_{H,K}}(B^{H,K},t)=t$ if

$$\Phi_{H,K}(x)= \biggl(x \biggm/\sqrt{2^{2-K} \log^{+}\log ^{+}\frac{1}{x}} \biggr)^{\frac{1}{HK}}, \quad x>0. $$

On the other hand, since Chung’s law and Strassen’s functional law of the iterated logarithm appeared, the functional law of the iterated logarithm and its rates for some classes of Gaussian processes have been discussed by many authors (see, for example, Csörgö and Révész [9], Lin *et al.* [10], Dudley and Norvaiša [7], Malyarenko [11]). However, almost all results considered only some Gaussian processes with stationary increments, and there has been little systematic investigation on other self-similar Gaussian processes (see, for example, Norvaiša [8], Tudor and Xiao [12], and Yan *et al.* [13]). The main reason for this is the complexity of dependence structures for self-similar Gaussian processes which do not have stationary increments.

Motivated by these results, in this paper, we consider the law of the iterated logarithm and Φ-variation of a sub-fractional Brownian motion. Recall that a mean-zero Gaussian process $S^{H}=\{S^{H}_{t},t\geq0\} $ is said to be a sub-fractional Brownian motion (in short, sub-fBm) with Hurst index $H\in(0,1)$, if $S^{H}_{0}=0$ and

1.2 $$ R_{H}(s,t):=E \bigl[S^{H}_{s}S^{H}_{t} \bigr] =s^{2H}+t^{2H}-\frac{1}{2} \bigl[(s+t)^{2H}+ \vert t-s \vert ^{2H} \bigr] $$

for all $s,t>0$. When $H=\frac{1}{2}$, this process coincides with the standard Brownian motion *B*. Sub-fBm was first introduced by Bojdecki *et al.* [14] as an extension of Brownian motion, and it arises from occupation time fluctuations of branching particle systems with Poisson initial condition. A sub-fBm with Hurst index *H* is *H*-self-similar, Hölder continuous, and it is long/short-range dependent. A process *X* is long-range dependent if $\sum_{n\geq\alpha}\rho_{n}(\alpha)=\infty$ for any $\alpha>0$, and it is short-range dependent if $\sum_{n\geq\alpha}\rho_{n}(\alpha)<\infty $, where $\rho_{n}(\alpha)=E[(X_{\alpha+1}-X_{\alpha})(X_{n+1}-X_{n})], \alpha>0$. However, when $H\neq\frac{1}{2}$, it has no stationary increments. Moreover, it admits the following (quasi-helix) estimates:

1.3 $$ \bigl[\bigl(2-2^{2H-1}\bigr)\wedge1\bigr] \vert t-s \vert ^{2H}\leq E \bigl[ \bigl(S^{H}_{t}-S^{H}_{s} \bigr)^{2} \bigr]\leq\bigl[\bigl(2-2^{2H-1}\bigr)\vee 1\bigr] \vert t-s \vert ^{2H} $$

for all $t,s\geq0$. More works on sub-fractional Brownian motion can be found in Bojdecki *et al.* [15, 16], Shen and Yan [17], Sun and Yan [18], Tudor [19, 20], Yan *et al.* [21, 22], and the references therein. For the above discussions, we find that the complexity of sub-fractional Brownian motion is very different from that of fractional Brownian motion or bi-fractional Brownian motion. Therefore, it seems interesting to study the iterated logarithm and Φ-variation of sub-fractional Brownian motion. In the present paper, our main objectives are to expound and to prove the following theorems.

### Theorem 1.1

*Let* $0< H<1$, *we then have*

1.4 $$ \limsup_{s\rightarrow0}\frac{ \vert S^{H}_{t+s}-S^{H}_{t} \vert }{\varphi _{H}(s)}=1, $$

*almost surely*, *for all* $t > 0$, *where the function* $\varphi_{H}$ *is defined by*

$$\varphi_{H}(s)=s^{H}\sqrt{2\log^{+} \log^{+} (1/s )},\quad s > 0 $$

*with* $\varphi_{H}(0)=0$, *where* $\log^{+}x=\max{\{1, \log x\}}$ *for* $x\geq0$.

### Theorem 1.2

*Let* $0< H<1$, *and let* $\Phi_{H}$ *be defined as above*. *Then we have*

1.5 $$ S_{\Phi_{H}}\bigl(S^{H},T\bigr)=T,\quad{\rm a.s.} $$

*for all* $T>0$.

As an immediate question driven by Theorem 1.2, one can consider the following asymptotic behavior:

1.6 $$ \phi(\delta) \bigl(S_{\Phi_{H}}\bigl(S^{H},T,\delta \bigr)-T \bigr)\longrightarrow {\mathscr {L}}, $$

as *δ* tends to zero, where ${\mathscr {L}}$ denotes a distribution, $\phi(\delta)\uparrow\infty$ ($\delta\to0$), and $S_{\Phi _{H}}(S^{H},T,\delta)$ is defined as follows:

$$S_{\Phi_{H}}\bigl(S^{H},T,\delta\bigr)=\sup \bigl\{ S_{\Phi_{H}}\bigl(S^{H},T,\kappa\bigr):\kappa\in{\mathscr {P}} \bigl([0,t]\bigr), \vert \kappa \vert \leq\delta \bigr\} . $$

We have known that when $H=\frac{1}{2}$, the sub-fBm $S^{H}$ coincides with a standard Brownian motion *B*. So, the two results above are some natural extensions to Brownian motion (see, for example, Csörgö and Révész [9], Dudley and Norvaivsa [7], Lin *et al.* [10]). This paper is organized as follows. In Sect. 2, we prove Theorem 1.1. In Sect. 3, we give the proof of Theorem 1.2.

## Proof of Theorem 1.1

In this section and the next section, we prove our main results. When $H=\frac{1}{2}$, the sub-fBm $S^{H}$ is a standard Brownian motion, and Theorem 1.1 and Theorem 1.2 are given in Taylor [4]. In this section and the next section, we assume throughout that $H\neq\frac{1}{2}$.

### Lemma 2.1

*Let* *μ* *be a centered Gaussian measure in a linear space* ${\mathbb {E}}$, *and let* $A\subset{\mathbb {E}}$ *be a symmetric convex set*. *Then we have*

2.1 $$ \mu(A+h)\leq\mu(A) $$

*for any* $h\in{\mathbb {E}}$.

Inequality (2.1) is called Anderson’s inequality (see, for example, [23]). It admits the following version:

- Let $X_{1},\ldots,X_{n}$ and $Y_{1},\ldots,Y_{n}$ both be jointly Gaussian with mean zero and such that the matrix $\{EY_{j}Y_{j}-EX_{i}X_{j},1 \leq i,j\leq n\}$ is nonnegative definite. Then we have
  2.2 $$ P \Bigl(\max_{1\leq j\leq n} \vert X_{j} \vert \geq x \Bigr)\leq P \Bigl(\max_{1\leq j\leq n} \vert Y_{j} \vert \geq x \Bigr) $$
  for any $x>0$.

We also will need the next tail probability estimate which is introduced (Lemma 12.18) in Dudley and Norvaiša [7].

### Lemma 2.2

(Dudley and Norvaiša [7])

*Let* ${\mathbb {B}}$ *be a Banach space*, *and let* $S\subset{\mathbb {B}}$ *be a compact set such that* $cS\subset S$ *for each* $c\in(0,1]$. *Assume that* $S(\delta_{0})\subset S$ *is closed for some* $0<\delta_{0}\leq1$ *and that*

$$S(\delta)=\frac{\delta}{\delta_{0}}S(\delta_{0}) $$

*for* $0<\delta\leq\delta_{0}$. *If* $Y=\{Y(t),{t\in S}\}$ *is a mean*-*zero continuous Gaussian process with a self*-*similar index* $\alpha\in (0,1)$, *then*

$$\sigma_{\delta}^{2}:= \sup\bigl\{ E\bigl[Y(t) \bigr]^{2}:t\in S(\delta)\bigr\} =(\delta/\delta_{0})^{2\alpha} \sigma_{\delta_{0}}^{2} $$

*for every* $\delta\in(0,\delta_{0}]$. *Moreover*, *for any* $\theta\in (0,1)$, *there is a constant* $C_{\theta}\in(0,\infty)$ *depending only on* *θ* *such that*

2.3 $$ P \Bigl(\sup_{t\in S(\delta)} \bigl\vert Y(t) \bigr\vert >x \Bigr)\leq C_{\theta }\exp \biggl\{ -\frac{\theta x^{2}}{2\sigma_{\delta}^{2}} \biggr\} $$

*for all* $\delta\in(0,\delta_{0}]$ *and* $x>0$.

The above result is Lemma 12.18 in Dudley and Norvaiša [7].

### Lemma 2.3

(Dudley and Norvaiša [7])

*Suppose that* $\{\xi_{k},k\geq1\}$ *is a sequence of jointly normal random variables such that* $E\xi_{k}=0$, ${\rm Var}(\xi_{k})=1$ *for all* $k\geq1$, *and*

$$\limsup_{n\to\infty}\max_{\substack{k,m\in(n,2n]\\k\neq m}}E[\xi_{k} \xi _{m}]< \theta\in \biggl(0,\frac{1}{2} \biggr), $$

*then we have*

$$\limsup_{n\to\infty}\frac{\xi_{n}}{\sqrt{2\log n}}\geq1-2\theta $$

*almost surely*.

The above result is Lemma 12.20 in Dudley and Norvaiša [7].

### Lemma 2.4

*Let* $H\in(0,\frac{1}{2})\cup(\frac{1}{2},1)$. *Then the functions*

2.4 $$ \rho_{H}(t,s):= \textstyle\begin{cases} (t+s)^{2H}-t^{2H}-s^{2H}, & \textit{if }H>\frac{1}{2},\\ t^{2H}+s^{2H}-(t+s)^{2H}, & \textit{if }H< \frac{1}{2}, \end{cases} $$

*with* $s,t\geq0$ *are nonnegative definite*.

By Kolmogorov’s consistency theorem, we find that there is a mean-zero Gaussian process $\zeta^{H}=\{\zeta^{H}_{t}, t\geq0\} $ such that $\zeta ^{H}_{0}=0$ and

$$E \bigl[\zeta^{H}_{t}\zeta^{H}_{s} \bigr]=\rho_{H}(t,s) $$

for all $t,s\geq0$.

### Lemma 2.5

*Let* $H\in(0,\frac{1}{2})\cup(\frac{1}{2},1)$ *and* $t>0$. *Denote* $X_{t}(s):=S^{H}_{t+s}-S^{H}_{t}$ *for* $s\geq0$. *Then we have*

2.5 $$ E\bigl[X_{t}(s)\bigr]^{2}\sim \textstyle\begin{cases} s^{2H}, & \textit{if }t>0,\\ (2-2^{2H-1})s^{2H}, & \textit{if }t=0, \end{cases} $$

*as* $s\downarrow0$, *where the notation* ∼ *denotes the equivalence as* $s\downarrow0$ *for every fixed* $t>0$, *and*

2.6 $$ \frac{E[X_{t}(u)X_{t}(v)]}{\sqrt{E[X_{t}(u)^{2}] \sqrt{E[X_{t}(v)^{2}]}}} \leq C_{H} \biggl[\biggl( \frac{u}{v}\biggr)^{H}+\biggl(\frac{v}{u} \biggr)^{H} - \biggl\vert \sqrt{\frac {u}{v}}-\sqrt{ \frac{v}{u}} \biggr\vert ^{2H} \biggr] $$

*for all* $u,v\geq0$, *and* $t>0$.

### Proof

Clearly, we have

$$\begin{aligned} E\bigl[X_{t}(s)\bigr]^{2}&=(2t+s)^{2H}+s^{2H}-2^{2H-1} \bigl[(t+s)^{2H}+t^{2H} \bigr] \\ &=(t+s)^{2H} \bigl((2-x)^{2H}+x^{2H} -2^{2H-1} \bigl(1+(1-x)^{2H} \bigr) \bigr) \end{aligned}$$

for all $s,t\geq0$, where $x=\frac{s}{s+t}$. An elementary calculus may show that

$$\lim_{x\downarrow0}\frac{1}{x^{2H}} \bigl\{ (2-x)^{2H}+x^{2H} -2^{2H-1} \bigl(1+(1-x)^{2H} \bigr) \bigr\} =1, $$

as $x\to0$, which implies that estimate (2.5) holds.

Given $t>0$. Consider the Gaussian process $\zeta^{H}$ with the covariance $\rho_{H}$ defined by (2.4). Then we have

$$\begin{aligned} &E \bigl( \bigl[\zeta^{H}_{t+u}-\zeta^{H}_{t} \bigr] \bigl[\zeta ^{H}_{t+v}-\zeta^{H}_{t} \bigr] \bigr)\geq0 \end{aligned}$$

for all $u,v\geq0$. To see that the inequality holds, we define the function on ${\mathbb {R}}^{2}$

$$(x,y)\mapsto G(x,y):=1+(1-x-y)^{2H}-(1-x)^{2H}-(1-y)^{2H} $$

with $(x,y)\in{\mathbb {D}}:=\{(x,y)| x,y\geq0,x+y\leq1\}$. Then, on the boundary of ${\mathbb {D}}$, we have

$$\begin{aligned} \textstyle\begin{cases} G(0,y)_{|_{0\leq y\leq1}}=0& (0< H< 1),\\ G(x,0)_{|_{0\leq x\leq1}}=0& (0< H< 1),\\ G(x,y)_{|_{x+y=1}}=1-(1-x)^{2H}-(1-y)^{2H}\geq x^{2H}-(1-y)^{2H}=0& (\frac{1}{2}< H< 1),\\ G(x,y)_{|_{x+y=1}}=1-(1-x)^{2H}-(1-y)^{2H}\leq x^{2H}-(1-y)^{2H}=0& (0< H< \frac{1}{2}). \end{cases}\displaystyle \end{aligned}$$

Moreover, the equations

$$\begin{aligned} \textstyle\begin{cases} \frac{\partial G}{\partial x}(x,y)=-2H \{ (1-x-y)^{2H-1}-(1-x)^{2H-1} \}=0,\\ \frac{\partial G}{\partial y}(x,y)=-2H \{ (1-x-y)^{2H-1}-(1-y)^{2H-1} \}=0 \end{cases}\displaystyle \end{aligned}$$

admit a unique solution $(x,y)=(0,0)$. Thus, we get

$$\min_{(x,y)\in{\mathbb {D}}}\bigl\{ G(x,y)\bigr\} =\min \bigl\{ G(0,0),G(0,y)_{|_{0\leq y\leq1}},G(x,0)_{|_{0\leq x\leq 1}},G(x,y)_{|_{x+y=1}} \bigr\} $$

and

$$\max_{(x,y)\in{\mathbb {D}}}\bigl\{ G(x,y)\bigr\} =\max \bigl\{ G(0,0),G(0,y)_{|_{0\leq y\leq1}},G(x,0)_{|_{0\leq x\leq 1}},G(x,y)_{|_{x+y=1}} \bigr\} , $$

which imply

$$G(x,y)\geq0,\quad(x,y)\in{\mathbb {D}} $$

for all $\frac{1}{2}< H<1$ and

$$G(x,y)\leq0,\quad(x,y)\in{\mathbb {D}} $$

for all $0< H<\frac{1}{2}$. It follows that

$$\begin{aligned} &E \bigl( \bigl[\zeta^{H}_{t+u}-\zeta^{H}_{t} \bigr] \bigl[\zeta ^{H}_{t+v}-\zeta^{H}_{t} \bigr] \bigr) \\ &\quad =(2t+u+v)^{2H}+2^{2H}t^{2H} -(2t+u)^{2H}-(2t+v)^{2H} \\ &\quad =(2t+u+v)^{2H}G \biggl(\frac{u}{2t+u+v},\frac{v}{2t+u+v} \biggr) \geq 0\quad\biggl(\frac{1}{2}< H< 1\biggr) \end{aligned}$$

and

$$\begin{aligned} &E \bigl( \bigl[\zeta^{H}_{t+u}-\zeta^{H}_{t} \bigr] \bigl[\zeta ^{H}_{t+v}-\zeta^{H}_{t} \bigr] \bigr) \\ &\quad = (2t+u)^{2H}+(2t+v)^{2H}-(2t+u+v)^{2H}-2^{2H}t^{2H} \\ &\quad =-(2t+u+v)^{2H}G \biggl(\frac{u}{2t+u+v},\frac{v}{2t+u+v} \biggr) \geq 0\quad\biggl(0< H< \frac{1}{2}\biggr) \end{aligned}$$

for all $u,v\geq0$, which imply

2.7 $$ \begin{aligned}[b] E\bigl[X_{t}(u)X_{t}(v) \bigr]&=E \bigl( \bigl[S^{H}_{t+u}-S^{H}_{t} \bigr] \bigl[S^{H}_{t+v}-S^{H}_{t} \bigr] \bigr) \\ &=\frac{1}{2} \bigl(u^{2H}+v^{2H}- \vert u-v \vert ^{2H} \bigr) -\frac{1}{2}E \bigl( \bigl[\zeta^{H}_{t+u}- \zeta^{H}_{t} \bigr] \bigl[\zeta ^{H}_{t+v}- \zeta^{H}_{t} \bigr] \bigr) \\ &\leq\frac{1}{2}\bigl(u^{2H}+v^{2H}- \vert u-v \vert ^{2H}\bigr) \end{aligned} $$

for all $u,v\geq0$ and $t>0$. Combining this with (1.3), we give estimate (2.6) and the lemma follows. □

### Lemma 2.6

*For* $0< H<1$, *we then have*

2.8 $$ \limsup_{s\rightarrow0}\frac{ \vert S^{H}_{t+s}-S^{H}_{t} \vert }{\varphi _{H}(s)}\geq1, $$

*almost surely*, *for all* $t > 0$.

### Proof

Let $\varepsilon\in(0,1)$ and $t>0$. We see that

2.9 $$ \begin{aligned}[b] \limsup_{s\downarrow0} \frac{ \vert X_{t}(s) \vert }{\varphi_{H}(s)}&\geq \limsup_{n\to\infty}\frac{ \vert X_{t}(r^{n}) \vert }{\varphi _{H}(r^{n})}\\ &=\limsup _{n\to\infty} \frac{ \vert X_{t}(r^{n}) \vert }{(r^{n})^{H}\sqrt{2\log(-n\log{r})}} \\ &=\limsup_{n\to\infty} \bigl\vert X_{t} \bigl(r^{n}\bigr) \bigr\vert \bigl(2\bigl(r^{n} \bigr)^{2H}\log (-n\log{r} ) \bigr)^{-\frac{1}{2}} \\ &=\limsup_{n\to\infty} \bigl\vert X_{t} \bigl(r^{n}\bigr) \bigr\vert \bigl(2\log nE \bigl[X_{t} \bigl(r^{n}\bigr)^{2} \bigr] \bigr)^{-\frac{1}{2}} \end{aligned} $$

for every $r\in(0,1)$, by the fact $\log (-n\log{r} )\sim\log n$ ($n\to\infty$).

Now, we verify that

2.10 $$ \limsup_{n\to\infty} \frac{ \vert X_{t}(r^{n}) \vert }{\sqrt{2(\log n)E [X_{t}(r^{n})^{2} ]}}\geq1- \varepsilon, $$

almost surely, for $r\in(0, 1)$ small enough. In fact, by Lemma 2.3 we only need to prove

2.11 $$ \limsup_{n\to\infty}\sup_{(k,m)\in D_{n}} \frac{E [X_{t}(r^{k})(X_{t}(r^{m})) ]}{ \sqrt{E [X_{t}(r^{k})^{2} ]} \sqrt{E [X_{t}(r^{m})^{2} ]}}< \frac {\varepsilon}{2} $$

for any $\varepsilon\in(0,1)$, where $D_{n}=\{(k,m) | k,m\geq n,k\neq m\} $. Some elementary calculations may show that the following inequalities hold:

$$x^{2H}+x^{-2H}-\bigl(x^{-1}-x\bigr)^{2H} \leq (1+2H )x^{2-2H}\quad\biggl(\frac{1}{2}< H< 1\biggr) $$

and

$$x^{2H}+x^{-2H}-\bigl(x^{-1}-x\bigr)^{2H} \leq2x^{2H}\quad\biggl(0< H< \frac{1}{2}\biggr) $$

for any $x\in(0,1)$. It follows from Lemma 2.5 that there is a real $r\in(0, 1)$ small enough such that

$$\frac{E[X_{t}(r^{k})X_{t}(r^{m})]}{\sqrt{E [X_{t}(r^{k})^{2} ]} \sqrt{E [X_{t}(r^{m})^{2} ]}}< \frac{\varepsilon}{2} $$

for each $k\neq m$, which implies that (2.11) holds and (2.10) follows with probability one. Combining this with the arbitrariness of $\varepsilon\in(0, 1)$, (2.9), and (2.10), we get that inequality (2.8) holds for all $t>0$. □

To prove Theorem 1.1, we now need to introduce the reverse inequality of (2.8), i.e.,

2.12 $$ \limsup_{s\to0}\frac{ \vert S^{H}_{t+s}-S^{H}_{t} \vert }{\varphi _{H}(s)}\leq1 $$

almost surely, for all $t > 0$. The used method is due to the decomposition (2.7), i.e.,

$$\begin{aligned} E \bigl[X_{t}(u)X_{t}(v) \bigr] ={}&E \bigl[ \bigl(S^{H}_{t+u}-S^{H}_{t}\bigr) \bigl(S^{H}_{t+v}-S^{H}_{t}\bigr) \bigr] \\ ={}&\frac{1}{2} \bigl(u^{2H}+v^{2H}- \vert u-v \vert ^{2H} \bigr) \\ &{}-\frac{1}{2} \bigl\vert (2t+u+v)^{2H}+2^{2H}t^{2H} -(2t+u)^{2H}-(2t+v)^{2H} \bigr\vert \end{aligned}$$

for all $u,v\geq0$ and $t>0$. Recall that a mean-zero Gaussian process $B^{H}=\{B^{H}_{t},t\geq0\}$ is said to be a fractional Brownian motion with Hurst index $H\in(0,1)$, if $B^{H}_{0}=0$ and

2.13 $$ E \bigl[B^{H}_{s}B^{H}_{t} \bigr] =\frac{1}{2} \bigl[s^{2H}+t^{2H}- \vert t-s \vert ^{2H} \bigr] $$

for all $s,t>0$. When $H=\frac{1}{2}$, this process coincides with the standard Brownian motion *B*. Moreover, for all $t>0$, the process $\{ B^{H}_{t+s}-B^{H}_{t},s\geq0\}$ also is a fractional Brownian motion with Hurst index $H\in(0,1)$. It follows that

2.14 $$ \begin{aligned}[b] &E\bigl[\bigl(B^{H}_{t+u}-B^{H}_{t} \bigr) \bigl(B^{H}_{t+v}-B^{H}_{t}\bigr) \bigr]-E\bigl[X_{t}(u)X_{t}(v)\bigr] =2^{-1}E\bigl[\zeta^{H}_{t+u}- \zeta^{H}_{t}\bigr] \bigl[\zeta^{H}_{t+v}- \zeta^{H}_{t}\bigr] \end{aligned} $$

for all $u,v\geq0$ and $t>0$. More works on fractional Brownian motion can be found in Biagini *et al.* [24], Hu [25] and Mishura [26], Nourdin [27], and the references therein.

### Proof of Theorem 1.1

Given $\varepsilon>0$ and $\gamma\in(0,1)$ such that

$$E\bigl[X_{t}\bigl(\gamma^{n}\bigr)\bigr]^{2} \leq(1+\varepsilon)\gamma^{2nH} $$

for each $n\geq1$. Then we have

$$\begin{aligned} \begin{aligned} P\bigl( \bigl\vert X_{t}\bigl(\gamma^{n}\bigr) \bigr\vert \geq(1+\varepsilon)\varphi_{H}\bigl(\gamma ^{n}\bigr) \bigr) &=2P\bigl(X_{t}\bigl(\gamma^{n}\bigr)\geq(1+ \varepsilon)\varphi_{H}\bigl(\gamma^{n}\bigr)\bigr) \\ &=2P \biggl(\frac{X_{t}(\gamma^{n})}{\sqrt{E[X_{t}(\gamma ^{n})]^{2}}}\geq\frac{(1+\varepsilon)\phi_{H}(\gamma^{n})}{\sqrt {E[X_{t}(\gamma^{n})]^{2}}} \biggr) \\ &=2 \int_{\frac{(1+\varepsilon)\phi_{H}(\gamma^{n})}{ \sqrt {E[X_{t}(\gamma^{n})]^{2}}} }^{\infty}\frac{1}{\sqrt{2\pi}}e^{-\frac{1}{2}x^{2}}\,dx \leq2\bigl[n\log(1/\gamma)\bigr]^{-(1+\varepsilon)} \end{aligned} \end{aligned}$$

by the fact

$$\frac{(1+\varepsilon)\varphi_{H}(\gamma^{n})}{\sqrt{E[X_{t}(\gamma ^{n})^{2}]}}\geq \frac{(1+\varepsilon)\varphi_{H}(\gamma^{n})}{\sqrt{(1+\varepsilon)\gamma^{2nH}}} =\frac{(1+\varepsilon)(\gamma^{n})^{2H}\sqrt{2\log(-n\log\gamma)}}{ \sqrt{(1+\varepsilon)\gamma^{2nH}}}. $$

It follows from the Borel–Cantelli lemma that

2.15 $$ \limsup_{n\rightarrow\infty}\frac{ \vert X_{t}(\gamma^{n}) \vert }{\varphi _{H}(\gamma^{n})}\leq1+\varepsilon $$

almost surely. Given $\varepsilon\in(0,1/2)$, let $\gamma\in(0,1)$ satisfy

$$2\bigl(\gamma^{-1}-1\bigr)^{H}\leq\varepsilon,\quad \gamma^{H}\geq(1+\varepsilon )/(1+2\varepsilon). $$

We now need to prove the estimate

2.16 $$ \lim_{n\to\infty}\sup_{\gamma^{n+1}\leq u\leq\gamma^{n}} \biggl\vert \frac {X_{t}(u)}{\varphi_{H}(u)}-\frac{X_{t}(\gamma^{n})}{ \varphi_{H}(\gamma ^{n})} \biggr\vert \leq2\varepsilon $$

almost surely. For all $n=1,2,\ldots$ and $s\in[0, 1]$, let

$$X_{t}^{n}(s):=X_{t}\bigl((1-s) \gamma^{n}\bigr)-X_{t}\bigl(\gamma^{n} \bigr)=S^{H}_{t+(1-s)\gamma ^{n}} -S^{H}_{t+\gamma^{n}} $$

and

$$Y_{t}^{n}(s):=B^{H}_{t+(1-s)\gamma^{n}}-B^{H}_{t+\gamma^{n}}. $$

Then, for all $\gamma\in(0,1)$ and $n\geq1$, $Y_{t}^{n}=\{ Y_{t}^{n}(s),{s\in[0,1]}\}$ also is a fractional Brownian motion which admits the same distribution as $\{B^{H}_{s\gamma^{n}},s\in [0,1] \}$.

On the other hand, by (2.14) and Anderson’s inequality (2.1), we have

$$\begin{aligned} &P \Bigl(\sup_{\gamma^{n+1}\leq u\leq\gamma^{n}} \bigl\vert X_{t}(u)-X_{t} \bigl(\gamma ^{n}\bigr) \bigr\vert \geq\varepsilon \varphi_{H}\bigl(\gamma^{n+1}\bigr) \Bigr) \\ &\quad =P \Bigl(\sup_{s\in[0,1-\gamma]} \bigl\vert X_{t}^{n}(s) \bigr\vert \geq\varepsilon \gamma^{(n+1)H}\sqrt{\log\bigl(-(n+1)\log \gamma\bigr)} \Bigr) \\ &\quad \leq P \Bigl(\sup_{s\in[0,1-\gamma]} \bigl\vert Y_{t}^{n}(s) \bigr\vert \geq \varepsilon\gamma^{(n+1)H}\sqrt{\log\bigl(-(n+1)\log \gamma\bigr)} \Bigr) \\ &\quad =P \Bigl(\sup_{s\in[0,\gamma^{n}(1-\gamma)]} \bigl\vert B^{H}(s) \bigr\vert \geq \varepsilon\gamma^{(n+1)H}\sqrt{\log\bigl(-(n+1)\log\gamma \bigr)} \Bigr)\equiv \Lambda_{n}(\gamma,\varepsilon) \end{aligned}$$

for all $\gamma,\varepsilon\in(0,1)$ and $n\geq1$. It follows from Lemma 2.2 with $Y_{t}=B^{H}_{t+\cdot}-B^{H}_{t}$, $S=[0, 1]$, $S(\delta)=[0,\delta]$, $0<\delta\leq1$, $\theta=1/2$, and $C= C_{1/2}$ that

2.17 $$ \begin{aligned}[b] \Lambda_{n}(r,\varepsilon)&\leq C\exp \biggl\{ -\frac{\theta(\varepsilon \gamma^{(n+1)H}\sqrt{\log(-(n+1)\log\gamma)})^{2}}{2\sup\{E \vert Y_{t}(s) \vert ^{2}: t\in S(\delta)\}} \biggr\} \\ &=C\exp \biggl\{ -\frac{\frac{1}{2}\varepsilon^{2}\gamma^{2(n+1)H} \log (-(n+1)\log\gamma)}{2[r^{n}(1-\gamma)]^{2H}} \biggr\} \\ &=C\exp \biggl\{ -\frac{\varepsilon^{2}\gamma^{2H}\log(-(n+1)\log\gamma)}{ 4(1-\gamma)^{2H}} \biggr\} \end{aligned} $$

for each $\gamma^{n+1}< e^{-e}$. Taking $\varepsilon\in(0,1/2)$ and $\gamma\in(0,1)$ such that

$$2\bigl(\gamma^{-1}-1\bigr)^{H}\leq\varepsilon,\quad \gamma^{H}\geq(1+\varepsilon )/(1+2\varepsilon) $$

to see that

$$\begin{aligned} \Lambda_{n}(\gamma,\varepsilon)&\leq C\exp \biggl\{ -\frac{\varepsilon ^{2}\log(-(n+1)\log\gamma)}{ (2(\gamma^{-1}-1)^{H})^{2}} \biggr\} \\ &\leq-C\exp \bigl\{ \log\bigl(-(n+1)\log\gamma\bigr) \bigr\} =\frac{C}{-(n+1)\log \gamma} \end{aligned}$$

by (2.17), which gives

$$\begin{aligned} &P \bigl(\sup\bigl\{ \bigl\vert X_{t}(u)-X_{t}\bigl( \gamma^{n}\bigr) \bigr\vert :r^{n+1}\leq u\leq \gamma^{n}\bigr\} \geq\varepsilon\varphi_{H}\bigl( \gamma^{n+1}\bigr) \bigr) \\ &\quad \leq-C\exp \bigl\{ \log\bigl(-(n+1)\log\gamma\bigr) \bigr\} =\frac{C}{-(n+1)\log \gamma}. \end{aligned}$$

Therefore, by the Borel–Cantelli lemma, we have that

2.18 $$ \limsup_{n\to\infty}\sup_{\gamma^{n+1}\leq u\leq\gamma^{n}} \frac{ \vert X_{t}(u)-X_{t}(\gamma^{n}) \vert }{\varphi_{H}(\gamma^{n+1})}\leq\varepsilon $$

almost surely. Noting that $\varphi_{H}$ is increasing, we see that

$$0\leq\frac{\varphi_{H}(\gamma^{n})}{\varphi_{H}(u)}-1\leq\frac{\varphi _{H}(\gamma^{n})}{\varphi_{H}(\gamma^{n+1})}-1=\gamma^{-H}\sqrt{ \frac{\log (-n\log\gamma)}{\log(-(n+1)\log\gamma)}}-1 $$

for $\gamma^{n+1}\leq u\leq\gamma^{n}$. It follows that

$$\limsup_{n\to\infty}\sup_{\gamma^{n+1}\leq u\leq\gamma^{n}} \biggl\vert \frac {\varphi_{H}(\gamma^{n})}{ \varphi_{H}(u)}-1 \biggr\vert =\gamma^{-H}-1\leq\frac {\varepsilon}{1+\varepsilon}, $$

by the choice of *γ*. Combining this with (2.15) and (2.18), we get (2.16). Finally, by (2.15) and (2.16), letting $\varepsilon\downarrow0$, we get (2.12) for all $t > 0$, and Theorem 1.1 follows. □

### Remark 2.1

From the proof of Theorem 1.1 we see that the idea came from the decomposition

$$S^{H}_{t}\stackrel{d}{=}B^{H}_{t}+ \zeta^{H}_{t} $$

for all $t\geq0$, where $\stackrel{d}{=}$ stands for the equal in distribution and $\zeta^{H}$ is a Gaussian process with the covariance $\rho_{H}$ defined by (2.4). Thus, for a self-similar Gaussian process, $G=\{G_{t},t\geq0\}$ admits the decomposition

$$G_{t}\stackrel{d}{=}B^{H}_{t}+\xi_{t} $$

for all $t\geq0$, where $\xi_{t}$ is a suitable Gaussian process. We can show that a similar limit theorem holds. However, for a different self-similar Gaussian process (weighted-fractional Brownian motion, bi-fractional Brownian motion, etc.) one needs to consider some concrete estimates.

## Proof of Theorem 1.2

In order to prove Theorem 1.2, we first give a lemma which extends the related result for Brownian motion.

### Lemma 3.1

*Let* $0< H<1$ *and* $t>0$. *Denote*

$${\mathbb {S}}_{t,\delta}=\bigl\{ (u,v)| u,v\in[0,t];0< u+v\leq\delta\bigr\} $$

*for* $\delta>0$, *then we have*

3.1 $$ \lim_{\delta\downarrow0} \biggl(\sup_{(u,v)\in{\mathbb {S}}_{t,\delta}} \frac{ \vert S^{H}_{t+u}-S^{H}_{t-v} \vert }{\varphi_{H}(u+v)} \biggr)=1 $$

*almost surely*.

### Proof

Given $t\geq0$ and denote $\delta_{0}=\min\{t,e^{-e}\}$. Define the function $\delta\mapsto D(\delta)$ by

$$D(\delta):=\sup_{(u,v)\in{\mathbb {S}}_{\delta_{0},\delta}}\frac{ \vert S^{H}_{t+u}-S^{H}_{t-v} \vert }{\varphi_{H}(u+v)} $$

for each $0 <\delta\leq\delta_{0}$.

By Theorem 1.1, we have known that $\lim_{\delta\downarrow 0}D(\delta)\geq1$ almost surely. We now need to give the upper bound of $D(\delta)$. Let $\varepsilon\in(0, \frac{1}{2})$ and

$$X_{t}(u, v):=S^{H}_{t+u}-S^{H}_{t-v} $$

for $u, v\in[0,\delta_{0}]$. Denote $\delta_{n}:=\exp\{-n^{1-\varepsilon }\}$, ${\mathbb {S}}_{n}:={\mathbb {S}}_{\delta_{0},\delta_{n}}$ and

$$E_{n}:=\bigl\{ \omega\in\Omega:\sup\bigl\{ \bigl\vert X_{t}(u,v,\omega) \bigr\vert :(u,v)\in {\mathbb {S}}_{n} \bigr\} \geq(1+2\varepsilon)\varphi_{H}(\delta_{n})\bigr\} $$

for all $n\geq8$. We need to handle $P(E_{n})$. To this end, we define the process

$$Y_{t}(u,v):=B^{H}_{t+u}-B^{H}_{t-v}, \quad u, v\in[0,\delta_{0}]. $$

Then, for any $u_{1}, v_{1},u_{2}, v_{2}\geq0$, we have

$$\begin{aligned} &E\bigl[Y_{t}(u_{1}, v_{1})Y_{t}(u_{2}, v_{2})\bigr]-E\bigl[X_{t}(u_{1}, v_{1})X_{t}(u_{2}, v_{2})\bigr] \\ &\quad =2^{-1}E \bigl[\zeta^{H}_{t+u_{1}}- \zeta^{H}_{t-v_{1}} \bigr] \bigl[\zeta ^{H}_{t+u_{2}}- \zeta^{H}_{t-v_{2}} \bigr] \end{aligned}$$

by (2.14), which implies that the matrix

3.2 $$ \begin{aligned}[b] (a_{ij})_{n\times n},\quad a_{ij}=E\bigl[Y_{t}(u_{i}, v_{i})Y_{t}(u_{j}, v_{j})\bigr]-E\bigl[X_{t}(u_{i}, v_{i})X_{t}(u_{j}, v_{j})\bigr] \end{aligned} $$

is nonnegative definite for any $u_{i},v_{i}\in[0,\delta_{0}], i=1,\ldots ,n$. It follows from inequality (2.2) that

$$P \Bigl(\sup_{(u,v)\in{\mathbb {S}}_{\delta_{0},\delta}} \bigl\vert X_{t}(u,v) \bigr\vert >x \Bigr)\leq P \Bigl(\sup_{(u,v)\in{\mathbb {S}}_{\delta_{0},\delta}} \bigl\vert Y_{t}(u,v) \bigr\vert >x \Bigr) $$

for all $\delta\in(0,\delta_{0}]$ and $x>0$. By (2.16) and Lemma 2.2 with $\theta=\frac{1}{1+2\varepsilon}$, we then have

$$\begin{aligned} P(E_{n}) &=P \Bigl(\sup_{(u,v)\in{\mathbb {S}}_{n}} \bigl\vert X_{t}(u,v,\omega) \bigr\vert \geq(1+2\varepsilon) \varphi_{H}(\delta_{n}) \Bigr) \\ &\leq P \Bigl(\sup_{(u,v)\in{\mathbb {S}}_{n}} \bigl\vert Y_{t}(u,v) \bigr\vert \geq (1+2\varepsilon)\varphi_{H}(\delta_{n}) \Bigr) \\ & \leq C_{\theta}\exp \biggl\{ -\frac{\theta[(1+2\varepsilon) \varphi _{H}(\delta_{n})]^{2}}{ 2\sigma_{\delta}^{2}} \biggr\} \\ &=C_{\theta}\exp \biggl\{ -\frac{(1+2\varepsilon)\varphi_{H}^{2}(\delta_{n})}{ 2\sigma _{\delta}^{2}} \biggr\} \\ & \leq C_{\theta}\exp \biggl\{ -\frac{(1+2\varepsilon)( \delta _{n}^{H})^{2}\cdot2\log(-\log\delta_{n})}{2(\delta_{n})^{2H}} \biggr\} \\ &=C_{\theta}\exp \bigl\{ -(1+2\varepsilon)\log(-\log{\delta_{n}}) \bigr\} =C_{\theta}n^{-(1-\varepsilon)(1+2\varepsilon)} \end{aligned}$$

for every $n\geq1$. It follows from the Borel–Cantelli lemma that there exists $n_{0}=n_{0}(\omega)$ such that

$$\sup_{(u,v)\in{\mathbb {S}}_{n}} \bigl\vert X_{t}(u,v) \bigr\vert \leq(1+2\varepsilon )\varphi_{H}(\delta_{n}) $$

almost surely, for each $n\geq n_{0}$, which implies that

$$\begin{aligned} D(\delta) &\leq\sup_{(u,v)\in{\mathbb {S}}_{\delta_{n},\delta}}\frac{ \vert S^{H}_{t+u}-S^{H}_{t-v} \vert }{ \varphi_{H}(u+v)} \\ &=\sup_{(u,v)\in{\mathbb {S}}_{n}} \biggl\{ \frac{ \vert X_{t}(u,v) \vert }{\varphi_{H}(u+v)}:(u,v)\in{\mathbb {S}}( \delta) \biggr\} \\ &\leq\sup_{m\geq n}\sup_{(u,v)\in{\mathbb {S}}_{m}} \biggl\{ \frac{ \vert X_{t}(u,v) \vert }{\varphi_{H}(u+v)}:(u,v)\in{{\mathbb {S}}_{m}\setminus {\mathbb {S}}_{m+1}} \biggr\} \\ &\leq\sup_{m\geq n}\sup_{(u,v)\in{\mathbb {S}}_{m}} \frac{ \vert X_{t}(u,v) \vert }{ \delta_{m+1}^{H}\sqrt{2\log(-\log\delta_{m})}} \\ &\leq\sup_{m\geq n}\frac{(1+2\varepsilon)\varphi_{H}(\delta_{m})}{ \delta_{m+1}^{H}\sqrt{2\log(-\log\delta_{m})}} \\ &=\sup_{m\geq n}\frac{(1+2\varepsilon)\delta_{m}^{H} \sqrt{2\log(-\log \delta_{m})}}{ \delta_{m+1}^{H}\sqrt{2\log(-\log\delta_{m})}} \\ &=(1+2\varepsilon)\sup_{m\geq n}\biggl(\frac{\delta_{m}}{\delta_{m+1}} \biggr)^{H}\longrightarrow 1+2\varepsilon\quad(n\to\infty) \end{aligned}$$

for $\delta\leq\delta_{n}$ and $\varepsilon\in(0,1)$, since $\frac {\delta_{m}}{\delta_{m+1}}\to1$, as $m\rightarrow\infty$. This completes the proof. □

Finally, at the end of this paper, we give the proof of Theorem 1.2. We will use the local law of the iterated logarithm (Theorem 1.1) for $S^{H}$ and the Vitali covering lemma to introduce the next inequality and its reverse:

3.3 $$ S_{\Phi_{H}}\bigl(S^{H},T\bigr)\geq T $$

for all $T>0$, where $\Phi_{H}$ is defined in Sect. 1.

### Proof of Theorem 1.2

Let $H\neq\frac{1}{2}$. We first show that inequality (3.3) holds. Given $\delta>0$. Let $0<\varepsilon<1$ and

$$\begin{aligned} E_{\delta}={}& \bigl\{ (t,\omega)| t\in[0,T],\omega\in\Omega, \\ &{}\Phi_{H} \bigl( \bigl\vert S^{H}_{t+s}( \omega)-S^{H}_{t}(\omega) \bigr\vert \bigr) >(1- \varepsilon)s{\text{ for some (rational) $s\in(0,\delta)$}} \bigr\} . \end{aligned}$$

Clearly, we have that there exists $\xi>0$ such that

$$\Phi_{H} \biggl(\biggl(1-\frac{\varepsilon}{2}\biggr)^{H} \varphi_{H}(v) \biggr)>(1-\varepsilon)v $$

for each $0< v<\xi$ since $\Phi_{H}$ is regularly varying of order $H^{-1}$ and is asymptotic to $\varphi_{H}^{-1}$ near zero. Therefore, by Theorem 1.1, for all $t\in(0,T]$ and $\delta\in(0,\xi)$, we have $P(\{\omega:(t,\omega)\in E_{\delta}\})=1$. It follows from the Fubini theorem that $P(\{m(E_{\delta})= T\})=1$ for each $0 < v<\xi$, where $m(\cdot)$ denotes the Lebesgue measure on $[0,T]$. Clearly, the set of all intervals $[t,t+s]$ with $t\in[0,T]$ and arbitrarily small $s>0$ is a Vitali covering of the set

$$E:=\bigcap_{0< \delta\leq1}E_{\delta}=\bigcap _{j=1}^{\infty}E_{1/j} $$

and $P(E)=1$. According to the Vitali lemma, we can choose a finite sub-collection ${\mathscr {E}}_{\delta}$ of intervals of length less than *δ* which are disjoint and have total length at least $T-\varepsilon$. Then

$$\begin{aligned} S_{\Phi_{H}}\bigl(S^{H},T,\kappa\bigr) &\geq\sum _{j}\Phi_{H}\bigl( \bigl\vert S^{H}_{t'_{j}+s_{j}}-S^{H}_{t'_{j}} \bigr\vert \bigr) \\ &>(1-\varepsilon)\sum_{j}s_{j} >(1- \varepsilon) (T-\varepsilon) \end{aligned}$$

almost surely, where $\kappa=\{t_{i},i=0,1,2,\ldots,n\}\in{\mathscr {P}}([0,T])$ with mesh $|\kappa|\leq\delta$ such that for each of the disjoint intervals $[t'_{j}, t'_{j}+s_{j}]$ from ${\mathscr {E}}_{\delta}$ with total length at least $T-\varepsilon$, there is some *i* with $t_{i-1}=t'_{j}$ and $t_{i}=t'_{j}+s_{j}$. Therefore, for each $\delta>0$ small enough, we obtain that

$$\sup\bigl\{ S_{\Phi_{H}}\bigl(S^{H},T,\kappa\bigr):\kappa\in{ \mathscr {P}}\bigl([0,T]\bigr), \vert \kappa \vert \leq\delta\bigr\} >(1- \varepsilon) (T-\varepsilon),\quad{\rm a.s.} $$

This shows that inequality (3.3) holds by taking *δ* and *ε* decreasing to zero.

Now, let us prove the reverse inequality of (3.3). Let $\varepsilon>0$. For any partition $\kappa=\{t_{i},i=0,1,2,\ldots,n\}\in {\mathscr {P}}([0,T])$, denote $\Delta_{i}=t_{i}-t_{i-1}$ and $\Delta _{i}S^{H}=S^{H}_{t_{i}}-S^{H}_{t_{i-1}}$ for $i=1,\ldots,n$, and let

$$\begin{aligned} &I_{1}:= \bigl\{ i\in\{1,\ldots,n\} : \Delta_{i}S^{H} \in [0,(1+\varepsilon)\varphi_{H}(\Delta_{i})) \bigr\} , \\ &I_{2}:= \bigl\{ i\in\{1,\ldots,n\} : \Delta_{i}S^{H} \in[(1+\varepsilon )\varphi_{H}(\Delta_{i}), A \varphi_{H}(\Delta_{i})) \bigr\} , \\ &I_{3}:= \bigl\{ i\in\{1,\ldots,n\} : \Delta_{i}S^{H} \in[A\varphi _{H}(\Delta_{i}),\infty) \bigr\} \end{aligned}$$

with

3.4 $$ A=\sqrt{8\bigl(4+1/(2H)\bigr)}e^{2H}, $$

and hence the sum $S_{\Phi_{H}}(S^{H},T,\kappa)$ can be divided into three sums of small, medium, and large increments. For $i\in I_{1}$, we will show that the sum of $\Delta_{i}$ is close to *T* if the mesh of *κ* becomes small enough, while for $i\in I_{2}\cup I_{3}$, the sum of $\Delta_{i}$ is negligible.

*Step I*. We estimate the sum

$$\sum_{i\in I_{1}}\Phi_{H}\bigl( \bigl\vert \Delta_{i}S^{H} \bigr\vert \bigr). $$

Since $\Phi_{H}$ is regularly varying of order $\frac{1}{H}$ and is asymptotic near zero to $\varphi_{H}^{-1}$, again, there is a real number $\eta(c,\varepsilon)>0$ such that

3.5 $$ \begin{aligned}[b] \Phi_{H}\bigl(c \varphi_{H}(v)\bigr)\leq \bigl(c^{1/H}+\varepsilon \bigr)v \end{aligned} $$

for any $0< v<\eta(c,\varepsilon)$ and $c>0$. Take $\delta_{1}:=\eta (1+\varepsilon)$. For a partition $\kappa=\{t_{i},i=0,1,2,\ldots,n\}\in {\mathscr {P}}([0,T])$ with mesh $|\kappa|<\delta_{1}$, we then obtain

3.6 $$ \begin{aligned}[b] \sum_{i\in I_{1}} \Phi_{H}\bigl( \bigl\vert \Delta_{i}S^{H} \bigr\vert \bigr) \leq\sum_{i\in I_{1}}\Phi_{H} \bigl((1+\varepsilon)\varphi_{H}(\Delta_{i})\bigr)) \leq(1+ \varepsilon)^{1/H}T+\varepsilon T. \end{aligned} $$

*Step II*. We estimate the sum

$$\sum_{i\in I_{2}}\Phi_{H}\bigl( \bigl\vert \Delta_{i}S^{H} \bigr\vert \bigr). $$

Let $\delta>0$ and denote

$${\mathbb {U}}_{\delta}(u,v)= \bigl\{ (t,\omega)\in[0,T]\times\Omega : \bigl\vert S^{H}_{t+u}(\omega)-S^{H}_{t-v}( \omega) \bigr\vert < (1+\varepsilon )\varphi_{H}(u+v) \bigr\} $$

for any $u,v\geq0$, $u+v\leq\delta$, and $v\leq t$. By Lemma 3.1, for every $t\in(0,T)$, we have

$$\lim_{\delta\to0}1_{{\mathbb {U}}_{\delta}}(t,\omega)=1 $$

with probability one. It follows from Fatou’s lemma and the Fubini theorem that

$$\lim_{\delta\rightarrow0} \int_{[0,T]}1_{{\mathbb {U}}_{\delta}}(t,\omega)\,dt=T $$

with probability one. Let $\Omega_{1}$ be a subset of Ω such that $P(\Omega_{1})=1$, and for every $\omega\in\Omega_{1}$, there exists $\delta _{2}(\omega)>0$ such that

$$m \bigl(\bigl\{ t\in[0,T]:(t,\omega)\notin{\mathbb {U}}_{\delta}(u,v)\bigr\} \bigr) = \int_{[0,T]}\bigl[1-1_{{\mathbb {U}}_{\delta}}(t,\omega)\bigr]\,dt\leq \varepsilon $$

for all $\delta\leq\delta_{2}(\omega)$. We choose $\delta_{2}(\omega)\leq \eta(A,\varepsilon)$. If a partition $\kappa\in{\mathscr {P}}([0,T])$ with $|\kappa|\leq\delta_{2}(\omega)$ such that there exists an interval $[t_{i-1},t_{i}]$ contains a point of ${\mathbb {U}}_{\delta_{2}(\omega)}$, then $i\in I_{1}$. So, the total length of such intervals is at least $T-\varepsilon$, and in particular,

$$\sum_{i\in I_{2}}\Delta_{i}\leq\varepsilon. $$

This shows that

3.7 $$ \begin{aligned}[b] \sum_{i\in I_{2}} \Phi_{H}\bigl( \bigl\vert \Delta_{i}S^{H} \bigr\vert \bigr) \leq\sum_{i\in I_{2}}\Phi_{H} \bigl(A\varphi_{H}(\Delta_{i})\bigr) \leq \bigl(A^{1/H}+\varepsilon \bigr)\sum_{i\in I_{2}} \Delta_{i} \leq A^{1/H}\varepsilon+\varepsilon^{2} \end{aligned} $$

by (3.5) with $c=A$, provided $|\kappa|\leq\delta_{2}(\omega)$.

*Step III*. We estimate the sum

$$\sum_{i\in I_{3}}\Phi_{H}\bigl( \bigl\vert \Delta_{i}S^{H} \bigr\vert \bigr). $$

We shall show that the sum also is small. Denote

$$S_{m,j}= \biggl[\frac{j}{2}e^{-m},\biggl(1+ \frac{j}{2}\biggr)e^{-m} \biggr] $$

and

$${\mathbb {V}}_{m,j}(A)= \Bigl\{ \omega\in\Omega : \sup _{t,s\in S_{m,j}} \bigl\vert S^{H}_{t}( \omega)-S^{H}_{s}(\omega) \bigr\vert \geq A\varphi _{H}\bigl(e^{-m-2}\bigr) \Bigr\} $$

for any integer number $m\geq3$ and $j=0,1,\ldots,j_{m}=[2Te^{m}]-1$, where $[x]$ denotes the integer part of *x* and *A* is defined by (3.4). The intervals $S_{m,j},j=0,\ldots,j_{m}$, overlap and cover $[0,T]$. Moreover, we have

3.8 $$ \begin{aligned}[b] & \bigl\{ i\in I_{3}( \omega):e^{-m-1}< 2\Delta_{i}\leq e^{-m} \bigr\} ^{\sharp}\leq5 \{j=0,1,\ldots,j_{m}:\omega\in V_{m,j} \} ^{\sharp}\equiv Z_{m}(\omega) \end{aligned} $$

for each $\omega\in\Omega$, where $E^{\sharp}$ denotes the number of elements in a set *E*. In order to bound $Z_{m}$, we need to estimate $P({\mathbb {V}}_{m,j}(A))$ for $j=0,\ldots,j_{m}$, and we show that one can replace $S^{H}$ by a fractional Brownian motion $B^{H}$ with Hurst index *H*.

Given $u\geq0$. Recall that $X_{u}(t)=S^{H}_{u+t}-S^{H}_{u}$ and $Y_{u}(t)=B^{H}_{u+t}-B^{H}_{u}$ for each $t\in[0,1]$, and

$$\begin{aligned} E\bigl[Y_{u}(t)Y_{u}(s)\bigr]-E\bigl[X_{u}(t)X_{u}(s) \bigr] =2^{-1}E\bigl[\zeta^{H}_{u+t}-\zeta ^{H}_{u}\bigr] \bigl[\zeta^{H}_{u+t}- \zeta^{H}_{u}\bigr] \end{aligned}$$

for all $s,t\in[0,1]$, where $\zeta^{H}$ is defined in Sect. 2. Hence, the matrix

$$(a_{ij})_{n\times n},\quad a_{ij}=E \bigl[Y_{u}(t_{i})Y_{u}(t_{j}) \bigr]-E\bigl[X_{u}(t_{i})X_{u}(t_{j}) \bigr] $$

with $t_{1},t_{2},\ldots,t_{n}\in[0,1]$ is nonnegative definite. It follows from inequality (2.1) that

$$P \Bigl(\sup_{t\in[0,\delta]} \bigl\vert X_{u}(t) \bigr\vert >x \Bigr)\leq P \Bigl(\sup_{t\in[0,\delta]} \bigl\vert Y_{u}(t) \bigr\vert >x \Bigr) $$

for all $0<\delta\leq1$ and $x>0$. Thus, by applying Lemma 2.2 with $S=S(1)=[0,1]$ and $\theta=1/2$ to the Gaussian process $Y_{u}$, and setting $u:=(j/2)e^{-m}, \delta:=e^{-m}$, we find that there is a constant $C>0$ such that

$$\begin{aligned} \begin{aligned} P\bigl({\mathbb {V}}_{m,j}(A)\bigr)&\leq P \Bigl(\sup _{t\in S_{m,j}} \bigl\vert X_{u}(t) \bigr\vert >(A/2) \varphi_{H}\bigl(e^{-m-2}\bigr) \Bigr) \\ &\leq P \Bigl(\sup_{t\in S_{m,j}} \bigl\vert Y_{u}(t) \bigr\vert >2\delta^{H}\sqrt {\gamma\log(m+2)} \Bigr) \leq C(m+2)^{-\gamma} \end{aligned} \end{aligned}$$

for all $m\geq3$ and $j=0,1,\ldots,j_{m}$, which implies that

$$EZ_{m}\leq10CTe^{m}m^{-\gamma}. $$

By the Borel–Cantelli lemma, there exists a set $\Omega_{2}\subset\Omega$ with probability one and some integer number $m_{1}(\omega)\geq3$ dependent only on $\omega\in\Omega_{2}$ such that

3.9 $$ Z_{m}(\omega)\leq e^{m}m^{2-\gamma}, \quad\forall m\geq m_{1}(\omega). $$

Moreover, by Corollary 2.3 in Dudley [28] and (1.3), we see that there is another set $\Omega_{3}\subset\Omega$ with probability one such that, for each $\omega\in\Omega_{3}$, there exists a finite constant $D(\omega)$ such that

3.10 $$ \bigl\vert S^{H}_{t}(\omega)-S^{H}_{s}( \omega) \bigr\vert \leq D(\omega) (t-s)^{H}\sqrt {-\log(t-s)} $$

for $0\leq s< t\leq e^{-1}$.

Let now $\omega\in\Omega_{2}\cap\Omega_{3}$ and $m_{2}(\omega)\geq m_{1}(\omega)$ satisfy

$$M(\omega):=\frac{1}{2}D(\omega)^{\frac{1}{H}}2^{\frac{1}{2H}}\sum _{m\geq m_{2}(\omega)}m^{-2}\leq\varepsilon. $$

Denote $\delta_{3}(\omega):=e^{-m_{2}(\omega)}$ and

$$\Lambda_{m}:= \bigl\{ i : e^{-m-1}< 2\Delta_{i} \leq e^{-m}, i\in\{ 1,2,\ldots,n\} \bigr\} . $$

Let $\kappa=\{t_{i},i=0,1,2,\ldots,n\}\in{\mathscr {P}}([0,T])$ with $|\kappa|\leq\delta_{3}(\omega)$ and for each $m\geq m_{2}(\omega)$. Then $[t_{i-1},t_{i}]\subset S_{m,j}$ for every *m* and some $j=0,\ldots ,j_{m}$, if $i\in\Lambda_{m}$. Combining this with (3.8), (3.9), and (3.10), we have

3.11 $$ \begin{aligned}[b] \sum_{i\in I_{3}} \Phi_{H}\bigl( \bigl\vert \Delta_{i}S^{H} \bigr\vert \bigr) &=\sum_{m\geq m_{2}(\omega)}\sum _{i\in I_{3}\cap\Lambda_{m}}\bigl( \bigl\vert \Delta_{i}S^{H} \bigr\vert \bigr) \\ &\leq\sum_{m\geq m_{2}(\omega)}Z_{m}(\omega) \Phi_{H} \bigl(D(\omega ) \bigl(e^{-m}/2\bigr)^{H} \sqrt{\log2+m+1} \bigr) \\ &\leq M(\omega)< \varepsilon \end{aligned} $$

for such a $\kappa=\{t_{i},i=0,1,2,\ldots,n\}\in{\mathscr {P}}([0,T])$.

Finally, for $\omega\in\Omega_{1}\cap\Omega_{2}\cap\Omega_{3}$, by taking $0<\delta\leq\min\{\delta_{1},\delta_{2}(\omega),\delta _{3}(\omega)\}$ by using (3.6), (3.7), and (3.11), we get

$$\begin{aligned} S_{\Phi_{H}}\bigl(S^{H},T,\kappa\bigr)&=\sum _{j=1}^{3}\sum_{i\in I_{j}}\Phi _{H}\bigl( \bigl\vert \Delta_{i}S^{H} \bigr\vert \bigr) \\ &\leq(1+\varepsilon)^{1/H}T+ \bigl(1+T+A^{1/H}+\varepsilon \bigr)\varepsilon \end{aligned}$$

for every partition $\kappa\in{\mathscr {P}}([0,T])$ with $|\kappa |<\delta(\omega)$. Thus, we have gotten the desired reverse inequality of (3.3) by arbitrariness of $\varepsilon>0$, and the theorem follows. □

## Results, discussion, and conclusions

In this paper, we give an iterated logarithm and Φ-variation for a sub-fBm by using some *precise estimations and inequalities*. It is important to note that the method used here is also applicative to many similar Gaussian processes.
